# Understanding the Effect of Internal and External Factors on Households’ Willingness to Sort Waste in Dammam City, Saudi Arabia

**DOI:** 10.3390/ijerph18189685

**Published:** 2021-09-14

**Authors:** Ossama Ahmed Labib, Latifah Manaf, Amir Hamzah Sharaai, Siti Sarah Mohamad Zaid

**Affiliations:** 1 Department of Environment, Faculty of Forestry and Environment, Universiti Putra Malaysia, UPM, Serdang 43400, Selangor, Malaysia; latifahmanaf@upm.edu.my (L.M.); amirsharaai@upm.edu.my (A.H.S.); mz_sarah@upm.edu.my (S.S.M.Z.); 2 Department of Environmental Health, College of Public Health, Imam Abdulrahman Bin Faisal University, Dammam 31441, Saudi Arabia

**Keywords:** solid waste management, waste sorting and recycling, psychological factors, awareness, market incentives, government facilitators

## Abstract

The acceleration of growth in the population in Saudi Arabia and the increase in municipal solid waste generation have caused a problem in Dammam city: an increase in solid waste production. Therefore, solid waste sorting is an important practice of municipal solid waste management. The main objectives in this research are understanding the effect of internal and external factors on household willingness in sorting waste in Dammam city and studying the attempts to construct a theoretical research model by adding market incentives, government facilitators, and awareness into the popular planned behaviour theory to explain residents’ waste sorting intentions. The data collection and analysis are based on the questionnaire study, which is based on the questionnaire survey data from 450 households in Dammam. This study revealed that social influence significantly predicts households’ willingness to sort and recycle, that is, to promote recycling. Additionally, the variable social influence has a significant but low influence on households’ willingness to sort and recycle. The result of the structural equation model shows that perceived behavioural control significantly predicts households’ willingness to sort and recycle waste. This finding is consistent with the theoretical expectation. Therefore, this research shows that attitude, social influence, perceived behavioural control, market incentives, government facilitators and awareness positively and significantly affect residents’ waste sorting intentions. Additionally, this research corroborates the discrepancy between internal and external variables.

## 1. Introduction

Waste segregation is crucial to curtailing the current waste management problems. In spite of its importance, little attention has been paid to exploring households’ behavioural intentions towards waste sorting. The rising urban population growth has caused a dramatic increase in municipal solid waste (MSW) generation, which was projected to increase from two billion tons estimated in 2016 to 3.5 billion tons within 30 years in east Saudi Arabia [1]. This has and will continue to cause serious environmental, health, and socioeconomic impacts, as a huge amount of land is used for waste disposal and storage, which consequently leads to air, soil, and underground water pollution [1,2]. The major contributing factors to the high increase in waste generation include rapid urbanisation, industrialisation, a change in consumption pattern and lifestyle, as well as the introduction of hazardous waste that is harmful to the public and environment [2].

One of the most efficient ways to solve the current problems of waste management is to sort the waste at its source of generation. However, only a small percentage of waste is sorted and recycled globally [3]. For example, Khalil et al. noted that in Nigeria, only 2% of waste generated is recycled. Similarly, in China, approximately three-quarters of the total waste generated is deposited directly into landfills, and 5% of waste is sorted and recycled, but it has been reported that a large percentage of the waste produced can be reused and recycled through waste sorting [1,4]. Sorting waste properly could remove approximately one-third of the waste moving to landfills and increase recycled, reused, and remanufactured materials [5]. On the other hand, the direct disposal of waste without segregation can cause serious waste of resources, as well as environmental and health effects [6]. Additionally, waste disposal without effective sorting can lead to underground water and soil damage, as well as disruption to the eco-system balance. Similarly, waste incineration without proper segregation threatens human health by polluting the air [7].

In view of the above negative effects of indiscriminate waste disposal without segregation, waste sorting is considered as a sustainable way to curtail the problems of solid waste management [8]. It is generally believed that the residential sector has long been a major source of waste generation [9,10]. Thus, addressing the waste management problem can only be achieved with the active participation of residents. Previous studies have reported a low level of household participation in waste sorting at the source [1,11,12]. Therefore, it is pertinent to investigate the factors influencing households’ waste sorting intention in order to encourage people to participate in daily waste sorting.

The government of Saudi Arabia is conscious of the need to provide a lasting solution for solid waste management issues in the country by investing heavily in the sector. In its 2017 national budget, the government allocated SR 54 billion to the general municipal services, including water drainage and waste disposal, to achieve its target [13,14]. One of the major targets of the government is to improve and encourage participation in solid waste recycling and disposal practices. Some of the efforts include the recent approval of new regulations for the MSWM integrated framework. The responsibility to ensure the successful implementation of the integrated framework was placed on the Ministry of Municipal and Rural Affairs [15,16].

## 2. Aim of the Study

Several attempts have been made to encourage residents to participate in solid waste sorting and recycling activities in different countries. One of the strategies was through providing incentives (e.g., monetary reward), especially in consumer recycling. However, in [14], it was posited that economic incentives can only be used to achieve short-term recycling participation. Internal incentives, such as socio-psychological factors, should also be considered as an effective means to increase long-term participation in waste sorting and recycling. Additionally, the provision of recycling facilities and programs that are targeted at creating awareness and enhancing people’s actions will greatly determine the success of waste separation and recycling goals [17,18].

Internal factors, such as attitude, awareness and perceived control, were reported to significantly affect residents’ willingness to participate in waste sorting and recycling [19,20]. However, while there are few studies that investigate the effect of external factors in influencing recycling behaviour [21]., there is a lack of literature that investigates the combined effect of internal (psychological) and external factors on residents’ waste sorting and recycling practice in one single model. Therefore, the objective of this study was to investigate the influence of socio-psychological and external factors on residents’ willingness to participate in sustainable waste handling practices in Dammam city, Saudi Arabia, as described in the following:

1. Assess the residents’ psychological and external factors regarding sustainable waste handling in Dammam city;
2. Compare the sustainable waste handling practices of different income level groups in Dammam city;
3. Investigate the relationships between independent variables and the willingness to participate in sustainable waste handling practices in Dammam city;
4. Determine the impact of the independent variables on the willingness of residents to participate in sustainable waste handling in Dammam city.

The research questions examined in our study, which aimed to assist in finding solutions to problems related to solid waste handling and the better participation of the population, are as follows:

1. What are the factors that significantly predict the willingness to handle sustainable waste at different income levels in Dammam city?
2. What is the relationship between psychological and external factors regarding sustainable waste handling in Dammam city?
3. What are the effects of psychological factors on residents’ willingness to participate in sustainable waste handling in Dammam city?
4. What are the levels of attitude, awareness, perceived behavioural control, social influences, and the willingness/intention among households in the resource sorting and recycling of sustainable waste?
5. What are the relationships between attitude, awareness, perceived behavioural control, social influences, market incentives, governmental facilitators, and willingness/intention among households in Dammam city?
6. What is the effect of independent variables such as attitude, awareness, perceived behavioural control, social influences, market incentives, and governmental facilitators on residents’ willingness to participate in sustainable waste handling in Dammam city?

## 3. Literature Review

The objectives of municipal solid waste management are either reducing or eliminating the adverse impacts of waste materials on human health and the environment to support and promote economic development and create a superior quality of life whilst keeping costs low and preventing waste build-up [22]. Several studies have indicated that much of the municipal solid waste is generated from developing countries (approximately 55–80%) by commercial or market areas (10–30%), with varying quantities from streets, industries, and institutions [23,24,25]. In recent decades, the management of solid waste has become a critical issue facing countries worldwide [26,27] but particularly in developing countries [28], such as Saudi Arabia, which face challenges in managing solid waste [29]. This problem has become alarming, especially in cities such as Dammam, in which landfilling is the primary method of solid waste management. Internal incentives, such as socio-psychological factors, should be considered as an effective means to increase long-term participation in waste sorting and recycling [30,31,32].

Positive changes in people’s attitudes and behaviours depend on understanding differences in the socio-psychological background of the individual, which influence their decision on whether or not to participate in waste sorting and recycling activities. People that are willing to reduce the environmental impacts of the waste they produce, through what they buy, how they deal with waste in their homes, and their belief in waste sorting and recycling, which would have a major impact on improving the environment, can only do so if there are facilitating conditions and proper policies that guide their actions and participation [33]. Therefore, a greater effort is required to motivate and enlighten people to understand and appreciate the importance of responsible and sustainable waste management practice and to manage their waste in a more sustainable manner by sorting waste at the source and recycling it [33].

The previous literature on household solid waste recycling has focused mainly on four aspects of recycling: determinants of waste recycling behaviour, the efficiency of waste recycling schemes, and the partnerships between formal and informal recycling sectors, with the majority focusing on the effect of psychological factors on recycling behaviour, such as attitude, awareness, and perceived control, which were reported to significantly affect residents’ willingness to participate in waste sorting and recycling [34,35,36]. However, while there are few studies that investigate the effect of external factors on influencing recycling behaviour [21], to enhance our theoretical and practical knowledge of the behavioural changes that lead to households’ participation in recycling, this study adopted the theory of planned behaviour as the theoretical foundation of this research, which has been widely applied in various pro-environmental behavioural studies. Due to the inadequacy of the theory in providing a comprehensive explanation of factors influencing residents’ participation in waste sorting and recycling, the present study tends to introduce and incorporate some external variables as possible contributing factors. The additional variables hypothesised to be influential in determining residents’ willingness to participate in sustainable waste handling practice include social influence, market incentives, and government facilitators [37].

## 4. Research Framework

**Figure 1:**
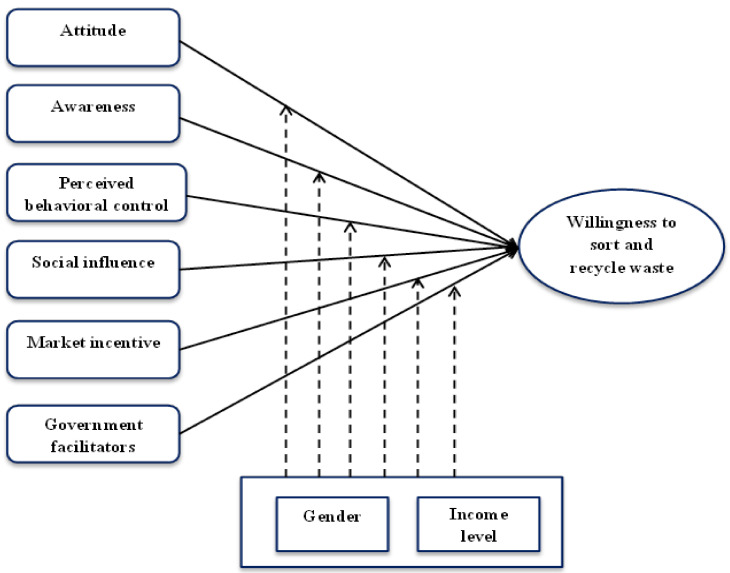
Research framework of the effects of different independent variables on the dependent variable (waste sorting intension).

This study focused on the self-reported residents’ sustainable waste handling practice. Previous studies on waste management behaviour and, more specifically, waste segregation and recycling behaviour formed the basis of this study, which focused on variables including attitude, perceived behavioural control, awareness, social influence, market incentives, government facilitators (as independent variables), and willingness/intention (as the dependent variable). As shown in Figure 1, the conceptual framework, which is based on the research evidence of studying a proposal model representing the relationship between internal/psychological factors (attitude, awareness, and perceived behavioural control), external factors (social influence, market incentives, and government facilitators) and residents’ sustainable waste handling practice (waste sorting and recycling).

### 4.1. Theory of Planned Behaviour

The theory of planned behaviour (TPB) posits that individuals first look at the likely consequences of the available alternatives (behavioural beliefs) when they are asked to decide on a course of action [38,39]. Second, they think about the normative expectations of people significant to them (normative beliefs), and then they weigh up the available resources at their disposal and potential obstacles that face them (control beliefs) [40,41]. These beliefs result in the formation of attitudes (a person’s overall assessment of the advantages and disadvantages of performing a given behaviour) towards the behaviour of interest, subjective norms (the perceived social pressure to engage or not to engage in a behaviour) concerning behaviour, and perceived behavioural control (people’s perceptions of their ability to perform a given behaviour) [42,43,44]. The TPB has also been widely used by many researchers on issues related to recycling behaviour; it examines the factors influencing people’s behaviour towards a particular issue [45,46,47,48].

### 4.2. Theory Model

The TPB asserts that when individuals are asked to decide on a course of action, they first look at the likely consequences of the available alternatives (behavioural beliefs); secondly, they consider the normative expectations of important people around them (normative beliefs); finally, they weigh up the available resources at their disposal and potential obstacles (control beliefs) [49,50]. These beliefs result, respectively, in the formation of attitudes (person’s overall assessment of the advantages and disadvantages of performing a given behaviour) towards the behaviour of interest, subjective norms (the perceived social pressure to engage or not to engage in a behaviour) with respect to behaviour, and perceived behavioural control (people’s perceptions of their ability to perform a given behaviour) [51]. However, perceived behavioural control (PBC) does not only predict behavioural intention but can also be used together with the intention to predict behaviour. As suggested by many researchers who applied the TPB model in their research, incorporating external variables improves the predictive ability of the TPB [52,53,54,55]. Similarly, Tonglet et al. (2004) suggest that future studies on waste sorting and recycling should include additional variables, such as past recycling behaviour and consequences of recycling and concern for the community [56]. Ajzen (1991) stated that TPB is “*open to the inclusion of additional predictors if it can be shown that they capture a significant proportion of the variance in intention or behaviour after the theory’s current variables have been taken into account*” [39,57,58,59].

One of the main challenges of the TPB is that the theory assumes an intention–behaviour correlation, which is mainly applicable when there is some level of volitional control [60]. The theory was developed to address situations that are not under volitional control. However, the intention and PBC as the direct determinants of behaviour in the TPB do not specifically address the normative and external influence, which may be important in explaining household waste sorting and recycling intention; hence, this study aimed to incorporate other variables from other theories into the TPB in understanding residents’ willingness to participate in waste sorting in Dammam city, Saudi Arabia.

Additionally, a study by Hu et al. (2018) revealed that attitude, subjective norms, and perceived behavioural control are all positively related to tourists’ intentions for waste reduction and recycling. Another study by Zhang et al. (2019) also indicated that attitude, subjective norm, and perceived behavioural control have a positive influence on residents’ intentions to participate in waste management activities and further noted that residents’ waste management intentions are the direct predictor of waste management behaviour [60,61,62].

### 4.3. Awareness

It was argued that individuals who are not aware of the detrimental effects of their actions towards the environment, or how they can positively avert the negative consequences of their actions, may not be likely to engage in pro-environmental activities [63]. The solution to the negative consequences of an individual’s actions often seems to lie in the provision of information and knowledge dissemination through learning and education. It was suggested that if individuals are aware of the issues, and how they can contribute to solving problems, they would change their behaviour accordingly. Individuals do not often feel a personal strong obligation to engage in a behaviour without being aware of the consequences of it [64]. When households are well informed about environmental needs and knowledgeable about how waste sorting helps to solve environmental problems, they are more likely to feel personally motivated and engage in recycling activities.

Previous studies revealed that awareness has a significant influence on pro-environmental behaviour [65,66]. Additionally, the findings of Chen and Tung (2010) and Xu et al. (2017) [9,10] proved that individuals’ awareness significantly predicts their willingness to participate in waste sorting. Additionally, Wang et al. (2017) indicated that customers’ electric vehicle (EV)-related awareness has a significant effect on their intentions to use EVs [67]. Hu et al. (2018) noted that tourists’ environmental awareness directly affects their willingness to participate in waste reduction activities in tourism areas suggested that contractor employees are more willing to reduce construction waste when they are sufficiently aware of construction waste [68].

Therefore, it can be proposed that when residents are fully aware of how to sort waste correctly and understand the positive outcomes of waste sorting and the adverse consequences of disposing of waste without sorting, their intentions to sort waste will be nurtured.

Additionally, when households are adequately aware of how to sort waste correctly, this reduces their perceived difficulties and improves their self-confidence in completing waste sorting behaviour. This is to say that residents’ awareness of waste sorting is positively correlated with perceived behavioural control [68].

### 4.4. Market Incentives

Market incentives refer to how residents obtain rewards through cash or any kind of incentive by selling various categories of recyclables materials to informal waste recyclers or collection companies. Similarly, residents can obtain other incentives/rewards from waste recycling companies through credit points, which can be exchanged for rewards [10]. However, some studies have reported that households’ waste separation and recycling behaviour can stop when monetary incentives end. This implies that placing a large amount of emphasis on only economic incentives to encourage behavioural changes may not be effective in the long term [9,14]. Therefore, it is crucial to examine the correlation between market incentives and residents’ willingness to participate in waste sorting. It was reported that monetary rewards can motivate non-recyclers to engage in waste separation and recycling. The monetary incentives can be collected at informal recycling centres, which serves as motivation to sort waste at the source and take it to recycling sites to obtain rewards [10].

Market-related factors, such as the informal recycling market, can increase the rates of waste sorting and recycling. In addition, a high number of collection sites within a residential area provided by government or private sectors may improve convenience for waste separation and recycling and ultimately boost residents’ willingness and participation. In many developing countries, studies have reported that the informal recycling sectors, which include waste pickers, waste material traders, itinerant buyers, and non-registered small-scale enterprises, have a great influence on the efficiency of household waste [69]. As reported by Nzeadibe (2009), residents who benefit from large monetary rewards from the informal sector and for whom the location of the recycling centre is convenient are more likely to perform waste separation in Nigeria. Therefore, the success of the informal recycling market will have a significant, direct effect on the residents’ waste sorting and recycling rates. This shows that market-related factors will significantly change residents’ waste separation and recycling behaviour in developing countries [10]. However, there is a lack of studies investigating the influencing factors from market-related factors to individual waste separation intention.

### 4.5. Government Facilitators

Government facilitators carry out public enlightenment and campaigns that can highlight the benefits and importance of waste separation and recycling, in addition to the provision of facilities for waste separation and recycling and situating them in a more convenient location for households. It was reported that the lack of waste separation and recycling facilities can prevent people from participating in the practice even if they are aware of the benefits of recycling [10]. Placing waste sorting and recycling facilities, such as drop-off centres, in a convenient location had a positive correlation with the increased recycling of waste other than newspaper. Therefore, placing recycling facilities near households increases residents’ willingness to participate in waste sorting and recycling [70].

Similarly, if the government provides recycling facilities in a convenient location for residents, it will lead to an increase in residents’ participation in waste sorting. Residents with highly perceived policy effectiveness would be more motivated to support government waste separation and recycling programs [71]. The residents’ perceived policy effectiveness can be improved by informing them about the efforts of the government towards solving waste management issues and the results or effects of their effort [20]. Government recycling policies that are aimed at improving residents’ waste separation and recycling education and the efforts to provide kerbside recycling facilities at a convenient location could also be supportive in the management of solid waste [72].

## 5. Research Methodology

### 5.1. Measurement

Various latent variables were included in the research framework (Figure 1). To measure these variables, instruments from previous studies of Khalil et al. (2018) and Xu et al. (2017) [9,10] were adapted. This was performed in order to ensure the reliability and validity of each latent variable. Modifications were made appropriately to suit the present research context. Respondents were asked to evaluate these items and show their opinion using a five-point Likert scale ranging from 1 (strongly agree) to 5 (strongly disagree).

### 5.2. Questionnaire Design and Pilot Test

This study designed a comprehensive questionnaire to comprehend residents’ waste sorting intentions. The instrument consists of three parts. The first part is the general information of the survey, such as informing the respondents that the survey will only be used for academic purposes, and their answers will be kept strictly confidential. The second part aims to collect the demographic information of the respondents. The last part is the studied variables and measurement items.

To ensure the reliability of the instrument, a pilot test was conducted with 50 residents in Dammam city. The results suggest that the test has high reliability and validity. Meanwhile, according to the survey feedback, some ambiguous items and improper statements were also revised. Finally, a formal questionnaire was formed. The final version of the measurement items of each variable is shown in Table 1.

### 5.3. Sample and Data Collection

The questionnaire survey was conducted in Dammam city, Saudi Arabia. Dammam is the capital of the eastern province of Saudi Arabia. The city contains the judicial and administrative bodies and many government departments of the eastern province. Dammam is the sixth-largest city in Saudi Arabia, after Riyadh, Jeddah, Mecca, Medina, and Taif, which makes it the largest city in the eastern province of the country. Dammam, similar to the other 12 regional capitals of Saudi Arabia, is not part of any governorate; instead, it is governed as a ‘municipality’ led by a mayor. Dammam is in the Dammam metropolitan area, which is popularly known as greater Dammam. In 2012, the population of the Dammam metropolitan area was estimated at 4,140,000, whereas the Dammam city will have a population ranging between 1.2 and 1.7 million in 2025. This rapid growth in population has an estimated annual growth rate of around 4.1% [13,73]. Dammam city is growing at a remarkably fast rate of 12% per year. This growth rate is seen as the fastest, in Saudi Arabia and among the Gulf Cooperation Council, as well as the Arab world. Greater Dammam emerged as the 4th largest area in both population and size in the Gulf Cooperation Council (GCC) in 2016. The data collection was conducted from 15 January to 15 February. In total, 450 households participate in the survey. Concerning the second objective, this section specifically discusses the perspectives of households towards the sustainable solid waste handling of residential areas for recycling practices in several distinct aspects. The following subsections show a descriptive discussion of the results obtained and an analytical discussion. Sampling successfully gathered 450 completed questionnaire sets among households in different districts in Dammam city, and the results of this research are presented based on the objectives of the study through the use of IBM SPSS version 22 (IBM Corp.: Armonk, NY, USA) and AMOS software. Cochran (1977) also developed a formula to estimate a representative sample size for analysis, which is shown below in Equation.

(1) $$Sample\;size=\begin{array}{c} z^{2}p(1-p)/e^{2}\\ 1+(z^{2}p(1-p)/e^{2}N) \end{array}$$

where no = z^2^ × p(1 − p)/e^2^, sample size = n0/1 + (n0)N, N population size, Z Z-score, e margin of error, and P standard of deviation.

The socioeconomic characteristics of the respondents are first presented, followed by a descriptive analysis of the objectives. Table 2 presents the demographic information of the respondents.

Table 2 shows the percentages of respondents for each gender in Dammam city. Of the respondents, 44% were females, and 56% were males. The total number of males was 252 and females was 198; therefore, at 44%, females are well represented in this study. The age group percentages of respondents in Dammam city are also shown. Respondents were divided into five age groups (Group 1: 18–29; Group 2: 30–39; Group 3: 40–49; Group 4: 50–59 and Group 5: 60+). The highest percentage of respondents were aged 40 to 49 years, with 209 respondents (46.4%), and the lowest percentage of respondents were aged 60 years and above, with 8 respondents (1.8%). The average age of the respondents was 34 years. For the distribution of educational level, respondents were divided into four groups (Group 1: primary school; Group 2: secondary school; Group 3: university degree, and Group 4: Msc/PhD). Most respondents had a college degree (74.7%), and many had an Msc/PhD educational qualification (21.5%). According to the questionnaire survey, high percentages (54.2%) of respondents were government employed; 2.25% and 16.9% were self-employed and private employed, respectively; 23.3% were unemployed; 0.45% were pensioners; 2.9% were housewives. Regarding the size of the households, 238% and 52.9% had 1 to 5 family members, respectively; 43.3% had 6 to 10 family members; only 3.8% had more than 10. Furthermore, the result indicated that the average household size was 6 persons, with a maximum of 13 persons and a minimum of 2 persons. Regarding the income distribution of the chief income earner of each household, 38.9% of the respondents, representing 175, had a monthly income above SR 5000, whereas 40% of the respondents, representing 180, had a monthly income between SR 1000 and SR 5000. However, only 95 respondents, representing 21.1%, had a monthly income below SR 1000.

## 6. Results and Discussion

### 6.1. Descriptive Statistics Analysis

This study utilised Pearson moment correlation analysis to establish relationships among the variables of the study. The result revealed that attitude has the highest significant and positive correlation with the willingness to sort and recycle waste (r = 0.731, *p* < 0.01). This result corroborates previous research that revealed that residents that have a positive attitude towards waste segregation and recycling are more likely to participate in the practice [2]. This is also consistent with some theoretical expectations [30,74] and the previous literature [22,75]. Some previous studies reported positive correlations between attitude and behavioural intention. However, the magnitude of the relationship depends on the behaviour under study, as posited by Schultz et al. (1995) [31]. In the waste separation and recycling literature, Bamberg et al. (2003) reported that a moderate but positive correlation is found between attitude and willingness to sort waste. This suggests that the attitude–willingness correlation for a particular behaviour may not necessarily be the same for another. For example, studies on energy-saving behaviour, green product purchasing behaviour, and recycling behaviour reported different levels of attitude–intention relationships [51,53,64,76]. Our findings show that attitude has a highly significant positive correlation with willingness to sort and recycle waste; this suggests that households who believe waste sorting is beneficial and rewarding may likely develop the willingness to sort waste. The majority of households in Dammam city, especially low-income households, believed that waste sorting and recycling are rewarding due to the financial benefits. This may likely influence their willingness and increase their attitude towards the practice.

The variable government facilitators have the second most significant positive correlation with the willingness to sort waste. This implies that the more access households have to facilities, the more likely they are to be willing to participate in waste sorting. This is consistent with the findings of Chen and Tung (2014) and Knussen and Yule (2008) [19,59]. Similarly, Wan et al. (2014) reported that the provision of recycling facilities explicitly motivates people to participate in recycling by improving their intention, even if they are not aware of the benefits of recycling. This means that facilitating conditions provide a means for households to engage in waste sorting and recycling, which may eventually become a habit if controlled.

These findings show that the market incentive has a direct association with the intention to sort waste. Besides its direct association with intention, market incentives are also associated with social influence and government facilitators. This implies that even if waste-sorting facilities are provided, the market for recycling is still an important stimulus for households to participate in sustainable waste-handling practices. Additionally, in Table 3 the result of the current study underlines the correlation between awareness and willingness to sort waste. As indicated by the result of the correlation analysis, awareness is associated with willingness and has a direct relationship with the perceived behavioural control and attitude towards waste sorting.

### 6.2. Structural Equation Model

Although other variables also have significantly predicted households’ willingness to sort and recycle, attitude appeared as the most important predictor of intention to sort waste. Consistent with the findings of this study, in a study conducted in the UK, Tonglet et al. (2004) reported that attitude was the most important predictor of recycling intention. The authors cited the availability of recycling facilities, high level of experience, and knowledge of the households as the main reasons for their findings. Thus, for residents that had low recycling abilities, perceived behavioural control would be a significant predictor of their willingness to sort waste. The findings of this research revealed that, in Dammam city, waste sorting and recycling facilities are not adequate, as people will be more motivated to participate in waste sorting if government facilitators, such as recycling facilities, are readily available; thus, this variable is the second most significant predictor of households’ intention to sort waste in this study. However, in low-income areas, market incentives appeared as strong predictors of households’ intention to sort waste. This is evident, as market incentive appeared as the third most important predictor of peoples’ intention to sort waste. These findings corroborated the findings of Knussen et al. (2004), who posited that in an area with relatively poor recycling facilities, attitude tends to be a significant predictor of recycling intention. Consistently, Ramayah et al. (2012) reported that attitude and social norms were significant predictors of recycling behaviour, while PBC did not influence intention to recycle in an area with poor waste management facilities in Malaysia. Additionally, a study by Karim Ghani et al. (2013), which investigated intention to sort food waste at the source among households living in an area with poor facilities, revealed that attitude was the only predictor of people’s intention, while other variables, such as social norms and perceived behavioural control, and situational factors did not predict willingness to sort and recycle.

Furthermore, the finding of the present study revealed that social influence significantly predicts households’ willingness to sort and recycle waste (β = 0.109, CR = 2.462, *p* = 0.012). This is consistent with the findings of Wan et al. (2014), who reported that recycling intentions are instinctively directed by social responsibility. The perceived social influence of important people, such as family and friends, plays a significant role in influencing individuals’ willingness to perform waste segregation and recycling practice. However, this type of normative influence would be more important at the early stage of the waste sorting and recycling program, the time when individuals would follow other peoples’ actions and their expectation about him/her to engage in the recycling program. Additionally, Huffman et al. (2014) also reported that when individuals perceived a strong social influence by the significant people around them to perform recycling, he/she would have an intention to recycle [77]. Wan et al. (2014) reported that strong social norms can decrease the influence of attitude on recycling intention [20]. This is to say that when there is social pressure to participate in waste sorting and recycling, individuals are likely to be influenced to participate, regardless of their level of attitude. In Dammam city, the variable social influence has a significant but low influence on households’ willingness to sort and recycle waste. Although the social influence positively affects households’ participation in waste sorting and recycling activities, disappointment in the waste management agencies for not providing the required recycling facilities in every part of the city discourages people from acting in that manner. This is consistent with the study of Wan et al. (2014) conducted in Bulgaria, which revealed that a lack of trust and fear of disappointment by waste management agencies have discouraged residents from participating in waste sorting and recycling.

Additionally, according to Table 4 the result of the structural equation model shows that perceived behavioural control also significantly predicts households’ willingness to sort and recycle waste (β = 0.131, CR = 2.619, *p* = 0.009). This finding is consistent with the theoretical expectation [27] but contrary with previous studies [78,79,80]. Our finding also supports that of Chaisamrej (2006) and Chu et al. (2013), who also reported that perceived behavioural control was the strongest predictor of waste sorting and recycling intention among college students in Thailand [81,82]. The ability of perceived behavioural control to significantly contribute to willingness to sort and recycle waste in our study was not unexpected since, although there are inadequacy facilities, most areas of Dammam city have at least some facilities for waste sorting. This was supported by the level of households’ government facilitators presented in the first objective of this study. Consistently, other studies investigating planned behaviour control (PBC) as a weak contributor to recycling intentions [1,19,83] also posited that when perceived control is low, the intention to participate in pro-environmental behaviour tends to be low. For this study, the possible explanation for the low contribution of perceived behavioural control on willingness to sort and recycle waste is that the study area lacks a formal recycling system, and recycling facilities and local collection areas did not cover every part of the city. This affects households’ perceived control, which consequently affects waste sorting and recycling activities in the study area. This is especially prevalent in high-income areas where households are more concerned about the availability of recycling facilities, which is directly linked to their perceived control over recycling. On the other hand, active recyclers, especially in low-income households, demonstrated high waste sorting intention, as some store their recyclable materials in their compound and subsequently take them to informal recycling companies for selling. In this process, the households’ recycling activities do not depend largely on the availability of recycling facilities and local collection areas provided by the waste management authorities. Nevertheless, providing satisfactory facilities and local collections in all the residential areas of Dammam city has the potential to increase the rate of waste sorting participation among households in the area. As reported by Ajzen (2011), the role of perceived control increases when there are external factors, such as facilities, the absence of which can affect people’s participation in any environmentally friendly behaviour [84].

Additionally, in Table 4 also, our findings revealed that awareness significantly influenced households’ willingness to sort and recycle waste with the regression weight (β = 0.362 CR, 5.366, *p* = 0.004). This finding corroborates those of previous studies (e.g., [19,75,84], reveals that awareness directly influences willingness to sort waste and is congruent with previous research that reported that the awareness of consequences or consideration of future consequences determines people’s recycling intentions [45,85]. The result that awareness is a significant predictor of intention to sort waste underlines the significance of enlightening people about the benefits of individual and collective waste sorting for themselves and others.

Moreover, the variable government facilitators were found to have a significant positive effect on households’ intention to sort waste. The prediction coefficient of government facilitators is β = 0.587, CR = 9.321, *p* = 0.001, which indicated that the greater the number of the government facilitators, the higher the willingness to sort waste. In previous studies, government facilitators were used together with the perceived behavioural control to represent an individual’s ability to engage in recycling. Additionally, government facilitators reflect the efficiency of solid waste management at the municipality level [86]. Consistent with our findings, most studies that used government facilitators revealed that a high level of government facilitators enables high participation in waste separation and recycling; consistently, having satisfactory recycling facilities strengthens people’s attitudes towards and participation in recycling [75,84,87]. This indicated the correlation between satisfaction with solid waste management services and the level of households’ participation in waste sorting and recycling activities.

## 7. Conclusions

Households’ waste sorting intention is of great importance in managing solid waste issues. Thus, it is imperative to explore residents’ waste sorting intention and behaviour and comprehend the formation processes. Based on the theory of planned behaviour, this research builds a theoretical research model by including market incentives, government facilitators, and awareness to explore residents’ waste sorting intention. The results show that residents’ attitudes towards waste sorting, social influence, perceived behavioural control, market incentives, awareness, and government facilitators are all positively and significantly associated with households’ waste sorting intention. On the basis of research results, suggestions and recommendations to prompt residents to sort waste in their daily lives are proposed.

Generally, this research is effective for understanding households’ waste sorting intention. Nevertheless, it is undeniable that there are several limitations in this research. First, the data were only collected in Dammam city. Though Dammam city is one of the major cities and shares some common characteristics with other cities, the economic development level, residents’ environmental awareness, and waste sorting level may be different from other cities. Thus, it is recommended to generalise the current research results to other research contexts. In future research, the survey data should be collected from more cities. Second, the respondents of this research are urban residents. To enrich the research results and further improve the generalisability of results, respondents from rural areas should also be included. Finally, limited variables were added to the TPB model to explore residents’ waste sorting intentions. Other variables, such as emotion, motivation, and perceived value, are not considered. Future research can consider these variables to extend current researched. 

This research attempts to construct a theoretical research model by adding market incentive, government facilitators and awareness into the popular Theory of Planned Behavior to explain resident’s waste sorting intention and also, it is effective for understanding households’ waste sorting intention. Nevertheless, it is undeniable that there are several limitations in this research. Firstly, the data is only collected in Dammam city. Though Dammam city is one of the major cities and shares some common characteristics with other cities, the economic development level, resident’s environmental awareness and waste sorting level may be different from other cities. Thus, it should be cautioned to generalize the current research results to other research context. In the following research, the survey data should be collected from more cities. Secondly, the respondents of this research are urban residents. To enrich the research results and further improve the generalizability of results, respondents from rural areas should also be included. Finally, limited variables have been added into TPB model to explore resident’s waste sorting intention. Other variables such as emotion, motivation and perceived value are not considered. Future research can consider these variables to extend current research. This research had added the data surveying by data collection and analysis based on the questionnaire. It based on the questionnaire survey data from 450 households in Dammam. This study is revealed that social influence significantly predicts households’ willingness to sort and recycle waste (β = 0.109, CR = 2.462, *p* = 0.012). Also, the variable social influence has significant but low influence on households’ willingness for sorting and recycling. Although the social influence positively affects households’ participation in waste sorting and recycling activities, disappointment in the waste management agencies for not providing the required recycling facilities in every part of the city discourage people from acting in that manner. The result of the structural equation model shows that perceived behavioural control significantly predict households’ willingness to sort and recycle waste (β = 0.131, CR = 2.619, *p* = 0.009). This finding is consistent with the theoretical expectation. So that this research shows that attitude, social influence, perceived behavioral control, market incentives, government facilitators and awareness are positively and significantly affected resident’s waste sorting intention. Additionally, this research corroborates the discrepancy between psychological factors as variables, and suggests that the relationships between some internal and external variables and recycling intention in different income level of the respondents. Market incentives may likely strengthen the effect of residents’ intention on their behavior. This study is useful to comprehend resident’s waste sorting intention and valuable to encourage residents to sort waste in daily lives. The collected survey responses were coded in an Excel sheet before data analyzing by using IBM SPSS software (Version 22), including coding, screening, and cleaning to treating missing data. Data screening and cleaning were conducted to ensure error-free data set and to detect possible outliers. The presence of outliers could be due to (1) incorrect data entry, (2) failure to specify missing-value codes in computer syntax, (3) not a member of the population, and (4) the distribution for the variable in the sampled population has more extreme values than a normal distribution.

## Figures and Tables

**Table 1:** Description of measurement items.

Variable	Measurement Item	Mean	SD	Skewness	Kurtosis	Loading
Attitude	AT1: Waste sorting is good.	3.42	1.025	−0.578	−0.069	0.818 ***
	AT2: In my opinion, waste sorting is useful.	3.42	1.031	−0.382	−0.222	0.924 ***
	AT3: I find the idea of waste sorting distasteful.	3.58	1.062	−0.617	−0.012	0.821 ***
	AT4: Waste sorting is rewarding.	3.34	1.024	−0.621	−0.021	0.851 ***
	AT5: Waste sorting is sensible.	3.44	1.029	−0.571	−0.031	0.921 ***
Waste sorting awareness	AWR1: I think I am sufficiently aware of the value of waste sorting.	2.84	0.722	0.366	−0.384	0.822 ***
	AWR2: I think I am sufficiently aware of how to correctly sort waste.	2.87	0.810	0.506	−0.478	0.899 ***
	AWR3: I think I am sufficiently aware of the negative effects of waste.	2.90	0.810	0.444	−0.539	0.893 ***
Social influence	SI1: My neighbours think that I should sort waste in my daily life.	3.03	0.682	0.120	−0.382	0.807 ***
	SI2: My family members want me to sort waste in my daily life.	3.14	0.705	0.063	−0.182	0.795 ***
	SI3: My relatives wish me to sort waste in my daily life.	3.18	0.723	−0.047	−0.396	0.987 ***
Perceived behavioural control	PBC1: I have the skills and abilities to sort waste in daily life.	3.02	0.657	0.138	−0.196	0.914 ***
	PBC2: I feel easy and convenient when sorting waste in my daily life.	3.10	0.672	0.177	−0.069	0.888 ***
	PBC3: I have confidence that if I want to sort waste in my daily life, I can do it.	3.16	0.682	0.033	−0.369	0.922 ***
Market incentives	MI1: The prices quoted by recycling waste collection companies are reasonable.	3.48	0.973	−0.393	−0.053	0.897 ***
	MI2: You will bring along those recycling waste to the surrounding collection station in order to sell/exchange for points.	3.35	0.962	−0.260	−0.112	0.908 ***
	MI3: I can easily sell potential recycling waste to recycling waste collection companies.	3.46	0.998	−0.489	0.042	0.909 ***
Government facilitators	GF1: Government, community-driven campaigns can clearly explain the benefits and importance of waste separation.	2.84	0.722	0.366	−0.384	0.822 ***
	GF2: Government, community-driven separation campaigns can effectively improve waste separation awareness of residents.	2.87	0.810	0.506	−0.478	0.899 ***
	GF3: The waste separation bins provided by the government provide a favourable and convenient environment for residents.	2.90	0.810	0.444	−0.539	0.893 ***
Waste sorting intention	INT1: I intend to sort recyclable waste in the near future.	3.50	0.909	−0.275	−0.155	0.853 ***
	INT2: I intend to sort hazardous waste in the near future.	3.55	0.956	−0.402	−0.049	0.862 ***
	INT3: I intend to sort kitchen waste in the near future.	3.54	0.967	−0.303	−0.215	0.857 ***

*** Correlation is significant at the 0.001 level (2-tailed). Note: ATT: attitude; AWNS: awareness; PBC: perceived behavioural control; INT: intention; MI: market incentive; SI: social influence; GF: government facilitators.

**Table 2:** Demographic information of respondents.

Variable(s)	Frequencies	Percentage
Gender		
Male	252	56
Female	198	44
Age/Age Group		
18–29	129	28.7
30–39	71	15.8
40–49	209	46.4
50–59	33	7.3
60 above	8	1.8
Marital Status		
Married	246	54.7
Single	201	44.7
Divorce	2	0.44
Other	1	0.22
Educational Level		
Primary	2	0.44
Secondary	15	3.33
Diploma	-	-
College degree	336	74.7
Msc/PhD	97	21.5
Employment		
Govt. Employed	244	54.2
Private Employed	76	16.9
Self Employed	10	2.22
Unemployed	105	23.3
Housewife	13	2.9
Pensioner	2	0.44
Household Size		
1–5 Persons	238	52.9
6–10 Persons	195	43.3
11–15 Persons	17	3.8
16 Persons and above	0	0
Household Monthly Income		
Below SR 1000	95	21.1
SR 1000–SR 5000	175	38.9
Above SR 5000	180	40

SR = Saudi Riyal.

**Table 3:** Correlation matrix of the independent variables and the dependent variable.

Variables	ATT	AWNS	PBC	MI	SI	GF	INT
ATT							
AWNS	0.166 **						
PBC	0.211 **	0.289 **					
MI	0.204 **	0.170 *	0.150 **				
SI	0.164 **	0.158 **	0.350 *	0.194 **			
GF	0.204 **	0.270 **	0.250 **	0.404 *	0.441		
INT	0.731 **	0.322 **	0.251 **	0.556 **	0.344 **	0.604 **	

** Correlation is significant at the 0.01 level (2-tailed). * Correlation is significant at the 0.05 level (2-tailed). Note: ATT: attitude; AWNS: awareness; PBC: perceived behavioural control; INT: intention; MI: market incentive; SI: social influence; GF: government facilitators.

**Table 4:** Unstandardised and standardised regression weight in the hypothesised path model.

Hypothesised Relationship	Unstandardised Regression Weight Estimate	SE	Standardised Regression Weight Estimate	CR	*p* Value
INT ← ATT	0.712	0.078	0.610	8.648	0.000
INT ← AWNS	0.412	0.072	0.362	5.366	0.004
INT ← PBC	0.186	0.071	0.131	2.619	0.009
INT ← MI	0.528	0.076	0.446	6.003	0.002
INT ← SI	0.288	0.033	0.109	2.462	0.012
INT ← GF	0.661	0.056	0. 587	9.321	0.001

Note: ATT: attitude; AWNS: awareness; PBC: perceived behavioural control; INT: intention; MI: market incentive; SI: social influence; GF: government facilitators. ← this symbol means the regression and significantly from independent variables to dependent variable as example.

## Data Availability

In this manuscript, the datasets used and/or analyzed during the current study are available from the corresponding author on reasonable request.
